# Physicochemical and sensory quality of breakfast cereals produced from flour blends of maize and pigeon pea

**DOI:** 10.1016/j.heliyon.2025.e42612

**Published:** 2025-02-10

**Authors:** Clement Chinedum Ezegbe, Smith G. Nkhata, Ekpeno Sunday Ukpong, Mary Chikodili Ezeh, Kalu Sunday Okocha, Bernard Femi Pedro

**Affiliations:** a Department of Food Science and Technology, Faculty of Agriculture, Nnamdi Azikiwe University, PMB 5025, Awka, Anambra State, Nigeria; b Department of Agriculture and Food Systems, Faculty of Life Sciences and Natural Resources, Natural Resources College, Lilongwe University of Agriculture and Natural Resources, P. O Box 143, Lilongwe, Malawi; c Department of Food Science and Technology, Madonna University, Akpugo Campus, Enugu State, Nigeria

**Keywords:** Protein, Mineral, Pigeon pea, Functional, Proximate composition

## Abstract

Breakfast cereals offer affordable, quick and convenient meal options. This study examined the proximate, techno-functional, mineral and sensory qualities of breakfast cereals produced from a flour blend of maize and pigeon peas. Maize flour was substituted with pigeon pea flour at 0 %, 10 %, 20 %, 30 %, 40 % and 50 % levels. The composite flours (100 g), sugar (25 g), salt (5 g) and water (50 ml) were mixed to form a paste, baked at 110 °C for 1 h then cooled. The proximate composition, techno-functional properties, minerals and sensory quality were evaluated. With increase in pigeon pea flour incorporation, there were increase in crude protein from 10.93 to 14.30 %, ash from 1.56 to 3.11 % while crude fibre from 1.25 to 3.37 %. In contrast, there was significant (p < 0.05) decrease in water absorption capacity from 361.30 to 181.83 %. With 50 % pigeon pea flour incorporation, calcium significantly increased from 27.95 to 44.55 mg/100g while iron and magnesium decreased from 3.92 to 2.16 mg/100g and 75.07 to 63.16 mg/100g, respectively. Breakfast cereal containing maize:pigeon pea flour in the ratio 80:20 and 70:30 generally had the highest mean sensory scores on a 7-point Hedonic scale. This suggests that maize flour can be substituted with pigeon pea up to 30 % without producing significant changes in the sensory quality.

## Graphical abstract

**Figure:**
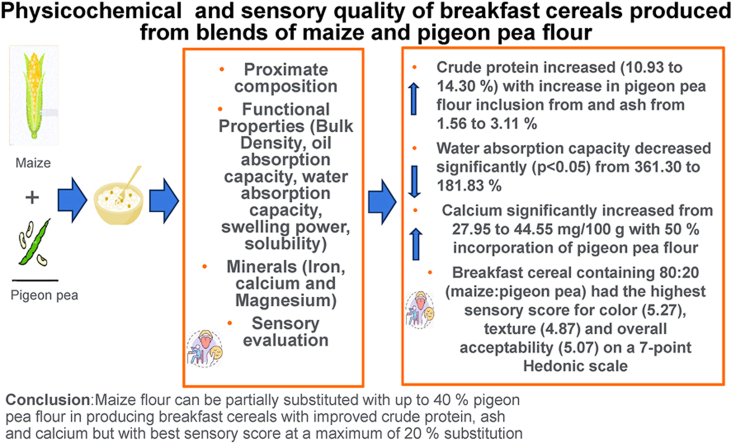

## 1 Introduction

Breakfast is taken as the nutritional foundation of the day and should supply about 300–500 kcal of energy [1]. Hence, skipping breakfast heightens the risk of encountering challenges with attentiveness, metabolism, and weight management, given its role as the key meal or nutritional cornerstone of the day [1]. Because of its ease of preparation, quick readiness, higher nutrient density, increased financial gain, and convenience, breakfast cereals are rapidly gaining acceptance, especially among urban dwellers [1].

Breakfast cereals are often consumed together with other complementary foods in order to have a balanced diet [2] which provides optimal energy levels, metabolic efficiency, and overall growth. An ideal breakfast meal should be rich in vitamins, fibre, iron and calcium [3]. The global breakfast cereal market is valued at $ 70 billion in 2024 and is projected to witness an annual growth rate of 4.1 % [3]. There is currently a greater innovative shift to ready-to-eat cereals (based on their ease of preparation), especially those rich in micronutrients, fiber and/or plant-based proteins with attractive but environmentally friendly packages [3].

It is recognised that essential amino acids such as lysine and tryptophan are crucial for body functions and are often deficient in most maize-based breakfast cereals [4]. Nonetheless, legume crops, are charcterized by their high sulfur-rich essential amino acids content, and are often used to complement cereals products to boost their nutritional quality [5]. Breakfast cereals fall into two categories: traditional (hot) cereals that need extra cooking or heating before they are consumed and ready-to-eat (cold) types which may not require further heating before consumption.

Maize (*Zea mays* L.), a grain, classified within Poaceae family, stands as one of the primary cereal crops consumed globally and provides good amounts of energy, minerals, carbohydrates, oils, and some macromolecules [6]. Tryptophan, lysine, threonine, valine, and sulfur amino acids have been shown to be limiting in maize [6]. Breakfast cereals produced with maize as a dominant component have high carbohydrate content and lack a balance of other essential nutrients. As a result, legumes, which are high in these amino acids and other nutrients are frequently added into breakfast cereals to boost the quality of the nutrients which help in reducing the incidence of deficiency diseases among susceptible populations [7].

Pigeon pea (*Cajanus cajan*) is a perennial legume crop, that belong to the Fabaceae family, and is classified as a rosid dicot [8]. Pigeon pea is used as a food crop and forage/cover crop, and is the principal accompaniment to rice or roti (flatbread) which is considered a staple diet throughout Africa [9]. Pigeon pea, combined with cereals, makes a well-balanced breakfast, and nutritionists recommend it as an important ingredient for producing a balanced diet [10]. Pigeon pea contains good amounts of macromolecules as well as essential amino acids such as methionine, lysine and tryptophan [9]. There is dearth of data and product made from the combination of maize and pigeon pea, hence this research aimed at developing breakfast cereals by utilizing combinations of flour blends of maize and pigeon pea in order to produce breakfast cereals that have not only increased nutrient content but also high consumer acceptability.

## 2 Materials and methods

### 2.1 Materials procurement

Yellow-coloured maize grains (8 kg) and cream-coloured pigeon pea (ICEAP 00040) (6 kg) seeds were acquired from Eke-Awka market Anambra State, Nigeria.

### 2.2 Raw materials for preliminary preparations

Before proceeding with further processing, maize grains and pigeon peas were sorted and thoroughly cleaned through hand-picking to remove stone, dirt, and chaff, as well as weevil-infected seeds and other foreign debris.

### 2.3 Processing maize grains into flour

The method outlined by Akubor et al. [11] was used to process maize flour with slight modification. The sorted maize grains were washed with potable water and boiled for 5 min. After boiling, the maize grains were drained and then dried in an oven (Gallenkamp 300 plus series, Cheshire, U.K.) at 60 °C for 10 h. The dried maize grains (9.54 %) were milled using an Attrition mill (9FC-36, China), sieved through a 150 μm sieve and the fraction that passed through the sieve was packaged and stored in an air-tight high-density polyethylene bags (Ziplock, China) and kept at room temperature (29 ± 2 °C) until needed.

### 2.4 Processing of pigeon peas into flour

The method described by Kaur et al. [12] for processing pigeon peas into flour was adopted in this research. After cleaning, the pigeon pea seeds were coarse-cracked using locally fabricated mill (Petroleum Motor spirit powered, tool steel blade/teeth, mild steel body, funnel shaped receptacle, belt pulley system). The cracked seeds were thoroughly blended with a wooden stirer along with 1 % cooking oil (Kings' vegetable oil) and allowed to stand for 3 h to aid in hull removal. The seeds were dried at 80 °C for 6 h and dehulling (through rubbing in between palms) was done to separate the hull from the dried seeds. Winnowing was used to separate the husks from the dehulled seeds, and the seeds were subsequently milled into a fine flour using a hammer mill. The flour was sieved (l mm) and the fraction that passed through the sieve was stored in an airtight bag and stored at room temperature (29 ± 2 °C) until needed.

### 2.5 Blend formulation

A Completely Randomized Design was adopted for this research to obtain the different proportions of maize and pigeon pea flour blends.The proportions of maize to pigeon pea flour in the composite flours were as follows: 100:0 (SMF1), 90:10 (SMF2), 80:20 (SMF3), 70:30 (SMF4), 60:40 (SMF5) and 50:50 (SMF6) respectively.

### 2.6 Breakfast cereal production

**Fig. 1:**
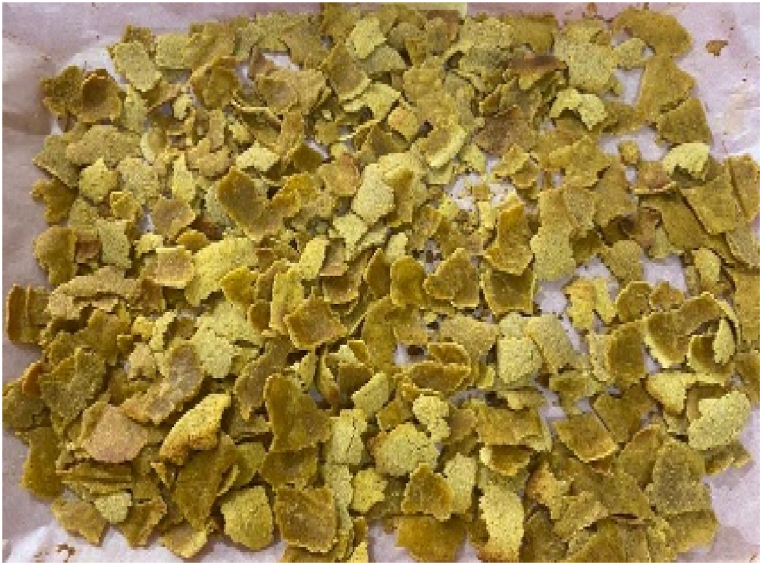
Breakfast cereal - 100 Maize: 0 Pigeon pea flour (SMF1).

**Fig. 2:**
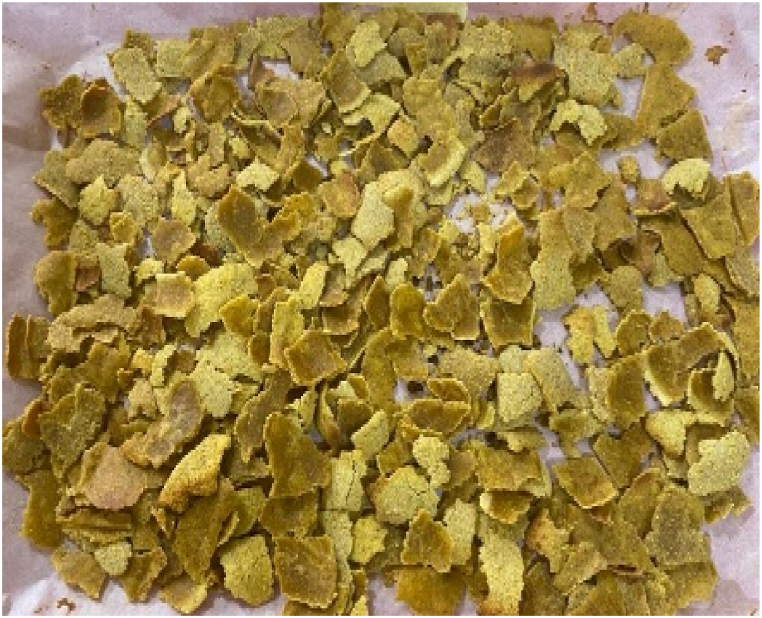
Breakfast cereal - 90 Maize: 10 Pigeon pea flour (SMF2).

**Fig. 3:**
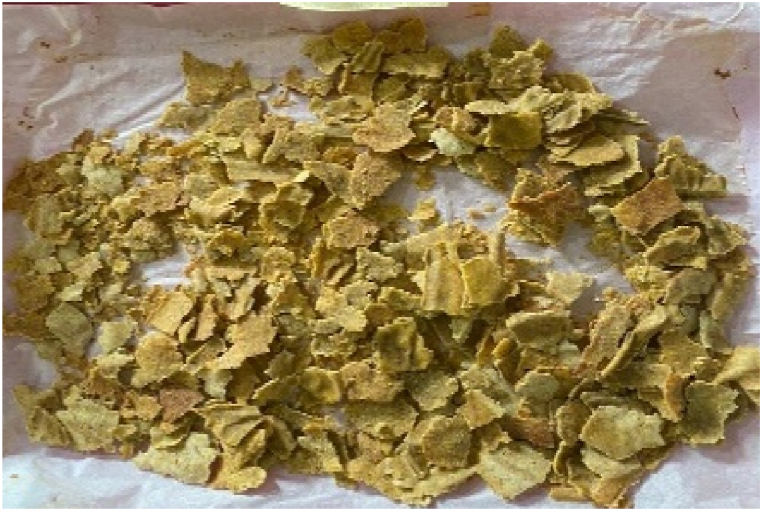
Breakfast cereal - 80 Maize: 20 Pigeon pea flour (SMF3)

**Fig. 4:**
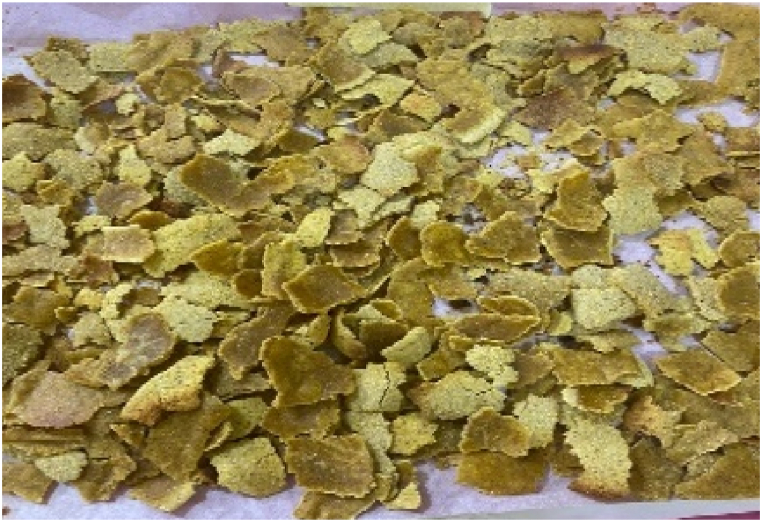
Breakfast cereal - 70 Maize: 30 Pigeon pea flour (SMF4).

**Fig. 5:**
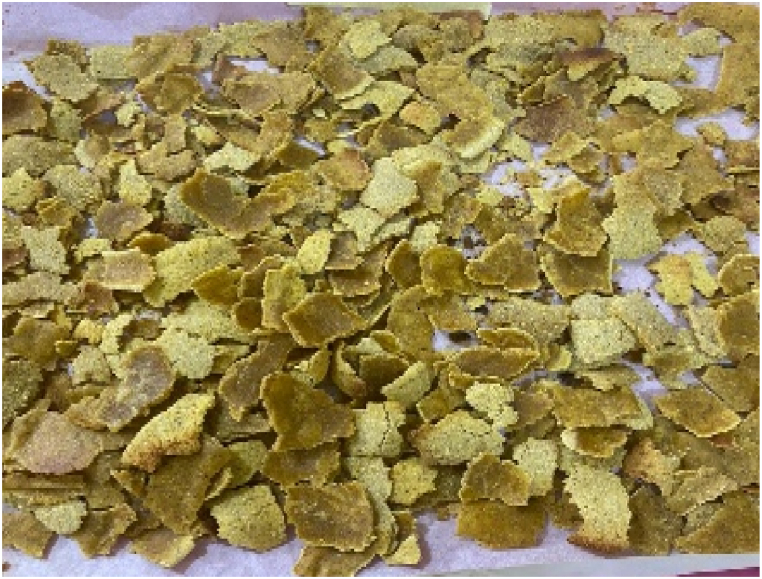
Breakfast cereal - 60 Maize: 40 Pigeon pea flour (SMF5).

**Fig. 6:**
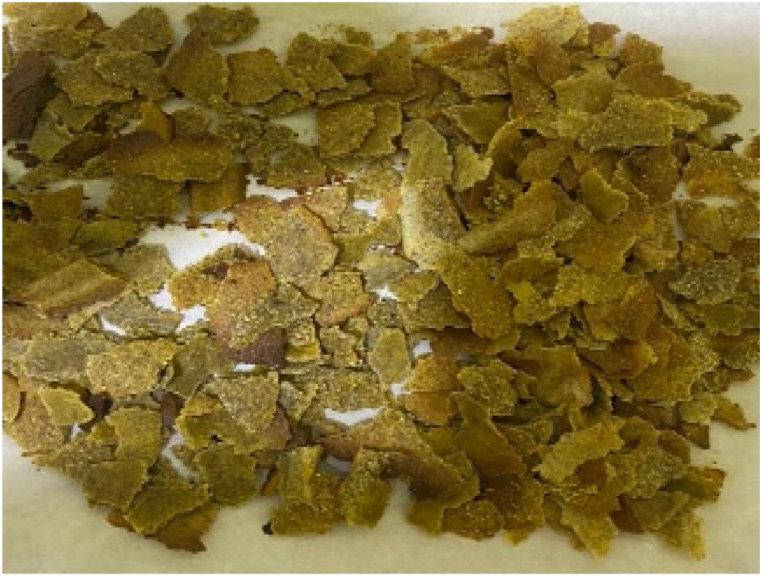
Breakfast cereal - 50 Maize: 50 Pigeon pea flour (SMF6).

The composite flour (100 g) was measured into a mixing bowl, followed by the addition of 25 g of sugar and 5 g of salt. Afterwards, 50 ml of water was included and mixed thoroughly to obtain a paste. The paste (8 mm) was placed on a parchment paper (Titan) which was placed on a tray. The paste was baked in an oven at 110 °C for 1 h to obtain a dry breakfast cereal. After cooling, the breakfast cereal samples (Fig. 1, Fig. 2, Fig. 3, Fig. 4, Fig. 5, Fig. 6) were manually broken (1 cm–2 cm) and packaged in high-density polyethylene bags for further analysis.

### 2.7 Proximate composition

The proximate composition of breakfast cereals was assessed using the method outlined by AOAC [13]. For determining moisture content, an oven drying method at 105 °C was employed using the Gallenkamp Oven (300 plus series) from Cheshire, United Kingdom, until a constant weight was attained (AOAC Method 925.10). Soxhlet extraction method using petroleum ether (Loba Chemie, Mumbai, India) was used for fat content determination (AOAC Method 920.39). Samples were incinerated at 550 °C for 5 h using a muffle furnace (Hasthas, Servell Engineers, Chennai, India) to assess ash content (AOAC Method 923.03). Crude fibre was determined by treating the sample with 2.5 % sulphuric acid, 2.5 % NaOH, ethanol, acetone, dried at 105 °C for 3 h and incinerated at 550 °C for 4 h (AOAC Method 962.09). The method of Kjeldahl was used in determining crude protein with a correction factor of 6.25 (AOAC Method 984.13). The carbohydrate content was determined using the difference method.

### 2.8 Determination of functional properties of maize, pigeon pea, and breakfast cereals

#### 2.8.1 Evaluation of bulk density

The breakfast cereal samples’ bulk density was evaluated with the method described by Onwuka [14]. Each sample was carefully filled into a 10 ml measuring cylinder of 1.5 cm diameter. After reaching the 10 mL mark, the cylinder was gently tapped on a laboratory bench until there was no further settling of the sample. The mass per unit volume (g/ml) was then calculated to determine the bulk density. Measurements were conducted in triplicate for each sample.

#### 2.8.2 Determination of water absorption capacity (WAC)

The method outlined by Onwuka [14] was used to determine the water absorption capacity (WAC). One gram of the sample was measured into a conical graduated centrifuge (Table Top TGL-16R) tube and mixed thoroughly for 30 s with 10 ml distilled water using a waring whirl mixer. The sample was allowed to stand for 30 min at room temperature before being centrifuged at 5000×*g* for 30 min. The supernatant was immediately removed from the graduated centrifuge tube:

1 $$\text{Water}\;\text{Absorption}\;\text{capacity}=\frac{\text{gram}\;\text{of}\;\text{water}\;\text{absorbed}\;\text{or}\;\text{retained}}{\text{gram}\;\text{of}\;\text{sample}}X\frac{100}{1}$$

#### 2.8.3 Swelling power and solubility determination

The evaluation of the swelling power and solubility of the breakfast cereal was conducted following the procedure outlined by Onwuka [14]. A quantity of 1 g of the breakfast cereal sample was carefully weighed into 15 ml centrifuge tubes and thoroughly mixed by manual shaking. The mixture was then heated in a water bath at 85 °C for 30 min, followed by cooling to room temperature and subsequent centrifugation at 2200 rpm for 15 min. The resulting clear supernatant was decanted into clean, dried Petri dishes whose weight had been pre-determined. The contents of the Petri dishes were evaporated at 100 °C in a hot air oven (Gallenkamp 300 plus series, Widnes, Cheshire, U.K.) for 30 min, after which they were removed, allowed to cool, and re-weighed. The difference in weight was used to calculate the mass of soluble compounds in the supernatant. The mass of each flour paste remaining in the centrifuge tubes was then recorded. The breakfast cereals sample's swelling power and solubility were calculated as follows:

2 $$\text{Swelling}\;\text{power}=\frac{\text{Mass}\;\text{of}\;\text{paste}\;}{\text{mass}\;\text{of}\;\text{sample}}$$

3 $$\text{Solubility}\;\left(\%\right)\frac{\text{Mass}\;\text{of}\;\text{soluble}\;\text{fraction}\;}{\text{mass}\;\text{of}\;\text{sample}}\times 100$$

### 2.9 Minerals determination procedure

Minerals (iron, calcium, and magnesium) were evaluated through the method used by Asaolu et al. [15]. Firstly, 2 g of the breakfast cereal was subjected to digestion with a 16 ml - mixture of nitric acid (conc.) and perchloric acid (conc.) in a 5:3 ratio and on a volume-by-volume basis. This digestion was done for 3 h at 80 °C in a water bath. Cooling was done, followed by filtration (0.45 μm) into a 100 ml standard flask, and it was then filled up to mark with distilled water. Specific appropriate lamps for the various minerals were fixed in the Spectrophotometer (Buck scientific model 211A, USA) accordingly. The digested samples were introduced into an air–acetylene flame to disintegrate the elements into atomic constituents. These constituents were then identified using a spectrophotometer at the appropriate wavelengths: 285.2 nm for magnesium, 248.3 nm for iron, and 422.7 nm for calcium. Concentrations of the minerals were determined through standard curves generated with standard solutions of 0.5, 1.0, 2.0, and 5.0 mg/l for the respective minerals manufactured by Sigma Chemical Company, United States of America. Similarly, blank solutions that did not contain the respective minerals were also subjected to the same procedure, and the values were deducted from those that contained the respective minerals.

### 2.10 Evaluation of sensory attributes

The formulated breakfast cereals were offered to 20 semi-trained panelists (10 males and 10 females, between 22 and 25 years, who consume breakfast cereals) made up of Nnamdi Azikiwe University Students. An informed consent was obtained from the panelists. A 7-point hedonic scale (where 1 represents dislike very much, 4 represents neither like nor dislike and 7 represents like very much) was used in evaluating the sensory quality of the samples. A sensory evaluation laboratory with well-lit boots was used. Randomly coded samples were given to the judges as well as potable water for mouth rinsing after evaluating each sample. Liquid peak milk (5ml/5 ml) was used in serving the breakfast cereals and afterwards aroma, taste, colour, texture, and overall acceptability of the samples were evaluated.

### 2.11 Statistical data analysis

The sensory scores obtained were then analysed using one-way analysis of variance (ANOVA) with Statistical Package for Social Sciences - version 25. Means separation was done using Duncan's Multiple Range Test at p < 0.05 level.

## 3 Results and discussion

### 3.1 Proximate composition of maize flour, pigeon pea flour and maize-pigeon pea breakfast cereal

#### 3.1.1 Moisture content

The moisture content of the samples is presented in Table 1. There was no significant difference (p < 0.05) in the moisture content between maize flour and pigeon pea flour. The moisture content of maize flour is 9.54 %, while pigeon pea flour is 9.84 %. The breakfast cereals have a moisture content range of 8.04–10.37 %. The breakfast cereal with 50 % maize and 50 % pigeon pea flours (SMF6) had the highest moisture content (10.37 %), while the breakfast cereal containing 100 % maize and 0 % pigeon pea flours had the least moisture content (8.04 %). The moisture content of breakfast cereals containing 60:40 % maize: pigeon pea flour (SMF5) and 50:50 % maize: pigeon pea flour (SMF6) differed significantly (p < 0.05). The moisture content of the breakfast cereal samples increased as the percentage of pigeon pea flour increased. The increase in moisture content could be due to high fiber content of pigeon peas which is capable of increasing the water retention capacity during baking. The moisture content of all samples, however, was within the standard limits, as the moisture threshold for flour-based products is 13 % [16].

**Table 1:** Proximate composition of corn flour, pigeon pea flour and breakfast cereals.

Samples	Maize: Pigeon Pea (%)	Moisture (%)	Crude Protein (%)	Ash (%)	Crude fiber (%)	Fat (%)	Carbohydrate (%)
Maize flour	–	9.54^bc^±0.34	12.87^bc^±0.44	1.10^a^±0.08	1.47^b^ ± 0.05	4.79^b^ ± 0.16	70.26^f^±0.89
Pigeon pea flour	–	9.84^c^±0.09	20.43^f^±0.18	3.16^g^ ± 0.10	4.12^h^ ± 0.06	2.62^a^±0.16	59.84^a^±0.06
SMF1	100:0	8.04^a^±0.04	10.93^a^±0.01	1.56^b^ ± 0.02	1.25^a^±0.07	6.19^f^±0.03	72.03^h^ ± 0.15
SMF2	90:10	8.13^a^±0.04	11.09^a^±0.06	1.69^b^ ± 0.02	1.74^c^±0.09	6.11^f^±0.01	71.27^g^ ± 0.21
SMF3	80:20	9.25^b^ ± 0.07	12.62^b^ ± 0.08	2.02^b^ ± 0.02	2.20 ^d^ ± 0.11	5.68^e^±0.02	68.24^e^±0.10
SMF4	70:30	9.29^b^ ± 0.15	13.07^c^±0.10	2.69^e^±0.02	2.51^e^±0.08	5.37^d^ ± 0.09	67.08^d^ ± 0.21
SMF5	60:40	9.86^c^±0.14	13.89^d^ ± 0.02	3.02^f^±0.02	2.94^f^±0.09	5.02^c^±0.02	65.29^c^±0.21
SMF6	50:50	10.37^d^ ± 0.05	14.30^e^±0.06	3.11^fg^ ± 0.03	3.37^g^ ± 0.02	4.79^b^ ± 0.12	64.08^b^ ± 0.22

Values are means ± standard deviation. Values with different superscripts in the same column differ significantly (p < 0.05).

Food with a moisture content exceeding 13 % is prone to yeast and mold growth, and also moisture-induced rancidity - hydrolytic [17]. The moisture content values recorded imply that the samples can be stored for extended period.

#### 3.1.2 Crude protein content

The maize flour had significanly lower crude protein content (12.87 %) compared to pigeon pea flour (20.34 %) (p < 0.05)

Crude protein content ranged from 10.93 % to 14.30 % in the breakfast cereal samples. Breakfast cereal containing the ratio of 50:50 for maize: pigeon pea flour (SMF6) had the highest protein level of 14.3 %, whereas breakfast cereal samples containing 100:0 % maize: pigeon pea flour (SMF1 - control) had the lowest protein content of 10.93 %. The crude protein content of breakfast cereal samples of 100 % maize: 0 % pigeon pea flour (SMF1 - control) and those with 90 % maize to 10 % pigeon pea flour (SMF2) did not differ significantly (p < 0.05) from each other. However, the crude protein content of the rest of the breakfast cereal samples differed significantly (p < 0.05). It was observed that the crude protein content of the breakfast cereal samples significantly increased as the amount of pigeon pea flour in the breakfast cereal samples was increased. Pigeon pea flour had higher crude protein content than maize flour and the increase in the crude protein content of the breakfast cereal samples is attributed to the increased proportion of pigeon pea suggesting that pigeon pea could be used to increase the protein content of maize-based breakfast cereals. In order to use pigeon pea to make up for the essential amino acid deficiencies in maize-based complementary foods, pigeon pea could be blended with maize. Combining cereals with legumes enhances the utilization of nutrients found in cereals [18]. Protein serves as a crucial building block for the body's integrity, which is important for development and the repair of worn-out tissues [18].

#### 3.1.3 Ash content

The ash content of maize flour (1.10 %) was significantly lower than that of pigeon pea flour (3.16 %) (p < 0.05).

The ash content of the breakfast cereals was in the range of 1.56 %–3.11 %. Breakfast cereals with 50:50 maize: pigeon pea flour (SMF6) had the highest ash content, whereas breakfast cereals containing 100:0 % maize: pigeon pea flour (SMF1 - control) had the lowest ash content of 1.56 %. The ash content of breakfast cereal samples containing 100:0 % maize: pigeon pea flour (SMF1-control), 90:10 – maize:pigeon pea flour (SMF2) and 80:20 % maize:pigeon pea flour (SMF3) were similar while the ash content of the rest of the samples differed significantly. The ash content of the breakfast cereals improved as the percentage of pigeon pea flour increased. The greater values for ash noted for the breakfast cereals produced in the research, particularly in SMF6 could be linked to the high mineral content in the pigeon pea. A - 93 % increase in the ash content (1.56–3.11 %) of breakfast cereals was achieved with the inclusion of pigeon pea. This increase seems very substantial for improving the mineral content of breakfast cereals and could be adopted in managing mineral deficiency in individuals.

#### 3.1.4 Crude fibre

Crude fibre content of the breakfast cereals were significantly different (p < 0.05) (Table 1). Pigeon pea flour had 4.12 % crude fiber which was significantly higher than that of maize flour (1.47 %). The breakfast cereals made from the blends had crude fibre range of 1.25 %–3.37 %. Breakfast cereal sample with 50:50 % maize: pigeon pea flour (SMF6) recorded the highest crude fibre content of 3.4 %, while the breakfast cereal sample with 100:0 % maize: pigeon pea flour (SMF1 - control) recorded the least (1.25 %). With decreasing amount of maize flour and an increasing amount of pigeon pea flour, the crude fibre content of the breakfast cereals significantly increased (p < 0.05) because pigeon pea flour had higher crude fibre content. The crude fibre content (1.25–3.37 %) in this research is lower than the crude fibre values of breakfast cereal produced from African yam bean and maize as recorded by Okafor and Usman [6]. This could be the result of the lower percentage (0–50 %) use of pigeon pea in this present research than that (50–100 %) reported by Okafor and Usman [6].

#### 3.1.5 Fat content

The amount of fat recorded for maize flour was significantly higher (4.79 %) than pigeon pea flour (2.62 %). The fat level of the breakfast cereals were in the range of 4.79 %–6.19 %. The breakfast cereal sample with 100 % flour (SMF1 - control) recorded the highest fat level (6.19 %), while the breakfast cereal with 50:50 % maize: pigeon pea flour (SMF6) contained the least quantity of fat (4.79 %). The fat content of breakfast cereal samples containing 100:0; maize flour:pigeon pea flour (SMF1 - control) and 90:10 – maize flour:pigeon pea flour (SMF2) were similar. However, the concentration of fat in breakfast cereal samples containing 80 % maize and 20 % pigeon pea (SMF3) and that of the sample that contained 70 % maize combined with 30 % pigeon pea flour (SMF4) differed significantly(p < 0.05). The fat contents of the breakfast cereals of 60:40 % maize: pigeon pea flour (SMF5) and 50:50 percent maize: pigeon pea flour (SMF6) were likewise significantly different (p < 0.05). The fat content of flour has an impact on the processing, packaging, and storage of flour-based products. As noted by Ezegbe et al. [18], dietary fat plays a crucial role in enhancing the absorption of fat-soluble vitamins, supplying essential fatty acids as well as volatile compounds crucial for flavour and sensory appeal. However, very high level of fat in foods that are baked can result in the development of rancidity during term storage. The fat content (4.79–6.19 %) obtained in this study is higher than the fat content (1.84–2.02 %) of breakfast cereal produced from African yam bean and maize as documented by Okafor and Usman [6]. The variation in the processing temperature could have influenced this change as the temperature of baking (110) in this research was lower than that (280 0C) used by Okafor and Usman [6]. Higher temperatures could result in the burning of more fats and other volatile components therein.

#### 3.1.6 Carbohydrate content

Maize flour had a significantly higher carbohydrate content of 70.26 %, compared to pigeon pea flour (59.84 %). The amount of carbohydrate in the various samples was in the range of 64.08–72.03 %. The breakfast cereal made from 100 % maize (SMF1), which served as the control had the highest carbohydrate content (72.03 %) while sample SMF6 which contains equal amounts of maize and pigeon pea flour (50:50 - maize:pigeon pea flours) had the lowest carbohydrate content (64.08 %). Notably, there was no significant variation in the amount of carbohydrate in the breakfast cereals made from 100 % maize flour (SMF1) and that made from 90 % maize and 10 % pigeon pea flour (SMF2) (p > 0.05). However, the carbohydrate content in breakfast cereal samples containing 80 % maize and 20 % pigeon pea flour (SMF3), and 70:30 maize: pigeon pea flour (SMF4) differed significantly. Similaely, the amount of carbohydrate in the samples that contained 60:40; maize:pigeon pea flours (SMF5) and 50:50 - maize:pigeon pea flour (SMF5) differed significantly (p < 0.05). The carbohydrate content of the breakfast cereals was reduced as the percentage of maize flour decreased. Maize has high carbohydrate content, which is consistent with the findings of this research which also shows that maize flour has the highest carbohydrate level. The carbohydrate content (64.08–72.03 %) of the breakfast cereals obtained from this research is higher than that obtained from the breakfast cereals made from African yam bean and maize (59.99–62.31 %) as highlighted by Okafor and Usman [6]. The higher carnohydrate content in pigeon pea, the difference in the processing steps and blend ratios would have contributed to this difference.

### 3.2 Functional properties of breakfast cereals

#### 3.2.1 Bulk density of breakfast cereals

Table 2 shows that the bulk density of the maize flour and pigeon pea flour significantly differed from each other (p < 0.05). The bulk density of maize flour was 0.80 g/cm^3^, while for pigeon pea flour was 1.03 g/cm^3^. The bulk density ranged from 0.83 g/cm^3^ (SMF6) to 0.85 g/cm^3^ (SMF1). Change in the quantity of pigeon pea flour included did not result in any significant (p < 0.05) effect on the bulk density of the breakfast cereals. This could have been the result of similar moisture contents of the flours (Table 1) as bulk density is affected by moisture content [19]. These results were lower than those reported by Bolade et al. [20], who recorded a bulk density range of 2.1–1.9 g/cm^3^ in a maize-based non-fermented breakfast cereal.

The bulk densities of the breakfast cereals in the present research were also lower than 4.95–5.24 g/cm^3^ recorded by Allai et al. [21] for breakfast cereals made from barley, corn, wheat, and Indian chestnut. Foods with higher bulk density occupy less space, allowing more products to be transported in a single shipment with better cost.

#### 3.2.2 Breakfast cereals water absorption capacity

Maize flour had significantly high water absorption capacity (WAC) of 231.10 %, compared topigeon pea flour with 188.50 % (Table 2). The range for WAC of the breakfast cereals is 181.83 %–361.30 % (Table 2). The breakfast cereal with 100:0 % maize: pigeon pea flour (SMF1 - control) recorded the highest WAC while the of 361.30 % 50:50 % maize: pigeon pea flour (SMF6) had the least WAC. It was observed that the WAC decreased as the percentage of maize flour decreased andpigeon pea flour increased. The observed trend in the WAC of the breakfast cereal may be due to an increase in protein content from pigeon pea flour which contained more protein. A food substance's water retention capacity is mostly determined by the water-binding ability of food ingredients [22]. The WAC of a food denotes the volume of water capable of being bound in each gram of flour which can influence food qualities such as moisture retention, texture, retrogradation and staling [22]. The reduction in the WAC as pigeon pea increased suggest the breakfast cereals were becoming dry or achieving a harder texture. Such products would have lower WAC and be less prone to moisture re-absorption during packaging and transportation, and consequently ensuring that the product reaches consumers with optimum texture.

**Table 2:** Functional properties of maize flour, pigeon pea flour and breakfast cereals from maize-pigeon pea flours.

Samples	Maize: Pigeon Pea	Bulk density (g/ml)	Water absorption capacity (%)	Swelling power (g/g)	Water Solubility index (%)
Maize flour	100:0	0.80^a^±0.01	231.10^e^±0.42	5.81^h^ ± 0.01	0.81^a^±0.02
Pigeon pea flour	0:100	1.03^b^ ± 0.01	188.50^b^ ± 0.71	1.38^a^±0.11	5.69^g^ ± 0.34
SMF1	100:0	0.83^a^±0.02	361.30^h^ ± 1.84	3.96^g^ ± 0.09	1.33^b^ ± 0.06
SMF2	90:10	0.83^a^±0.02	306.80^g^ ± 1.84	2.95^f^±0.07	1.89^c^±0.01
SMF3	80:20	0.83^a^±0.02	279.16^f^±1.21	2.23^e^±0.11	2.05^c^±0.06
SMF4	70:30	0.84^a^±0.01	217.11^d^ ± 2.85	1.99^d^ ± 0.02	2.41^d^ ± 0.06
SMF5	60:40	0.84^a^±0.01	203.98^c^±0.59	1.76^c^±0.05	2.95^e^±0.08
SMF6	50:50	0.85^a^±0.01	181.83^a^±2.08	1.58^b^ ± 0.03	3.28^f^±0.04

Values are means ± standard deviation of triplicates. Values in the same column with different superscripts are significantly (p < 0.05) different.

#### 3.2.3 Swelling power of breakfast cereals

Maize flour had significantly higher (p < 0.05) swelling power (5.81 g/g) compared to pigeon pea flour(1.38 g/g) (Table 2). The breakfast cereals had swelling power value in the range of 1.58 g/g to 3.96 g/g. The breakfast cereal with 100:0 - maize: pigeon pea flour (SMF1 - control) recorded the highest swelling power of 3.96 g/g, while 50:50 % maize: pigeon pea flour cereal (SMF6) hadleast swelling power of 1.58 g/g and there were significant differences among samples. As the amount of pigeon pea flour was increased, the swelling power decreased. The decrease in the swelling power of the breakfast cereals as the amount of flour from pigeon pea increased could be due to high protein content of pigeon peas compared to maize. Protein which interact with starch there by inhibiting its ability to swell when water is absorbed [22].

#### 3.2.4 Water solubility index of breakfast cereal samples

The water solubility index (WSI) of maize flour was significantly lower (0.81 %) compared to pigeon pea flour was (5.69 %) (Table 2). The breakfast cereals had WSI range of 1.33 %–3.28 %. Breakfast cereal sample with 50:50 % maize: pigeon pea flour (SMF6) recorded the highest solubility index of 3.28 %, while 100:0 % maize: pigeon pea flour (SMF1 - control) recorded the least solubility of 1.33 % and were significantly different among the six breakfast cereals. The WSI increased significantly as the amount of maize flour decreased and the amount of pigeon pea flour increased. Solubility expresses the ability of a solid in dissolving or dispersing in aqueous solutions such as water [23]. The increase in the breakfast cereal's WSI when the maize flour reduced could be due to its greater ease of dissolving in water [23]. The WSI of the breakfast cereals produced in this study is lower than that previsouly reported of 46–48 % produced from maize, barley, wheat and Indian horse chestnut [21]. The relatively **higher protein content, more complex and less soluble starches** in pigeon peas might partly explain the lower WSI observed in this study.

### 3.3 Selected minerals content of breakfast cereals produced with flours from maize and pigeon pea

The minerals content of the breakfast cereals produced from maize-pigeon pea flour blends is presented in Table 3. The iron content of the breakfast cereals varied from 2.16 mg/100g to 3.92 mg/100g.

**Table 3:** Mineral composition of Breakfast cereals from maize-pigeon pea flours.

Sample	Maize: Pigeon pea	Iron content (mg/100g)	Calcium content (mg/100g)	Magnesium content (mg/100g)
SMF 1	100:0	3.92^d^ ± 0.29	27.95^a^±0.07	75.07^e^±0.05
SMF 2	90:10	3.18^c^±0.04	29.52^b^ ± 0.73	70.56^d^ ± 0.12
SMF 3	80:20	3.01^c^±0.02	29.9^b^ ± 0.14	68.21^c^±0.01
SMF4	70:30	3.01^c^±0.04	33.74^c^±0.76	65.48^b^ ± 0.25
SMF5	60:40	2.63^b^ ± 0.07	40.81^d^ ± 0.01	65.33^b^ ± 0.01
SMF6	50:50	2.16^a^±0.06	44.55^e^±0.07	63.16^a^±0.01

The values in the table are means of three replicates ± standard deviation. Values in the same column with different superscripts differ significantly at 5 % probability level.

The breakfast cereal with 100:0 - maize:pigeon pea flour (SMF1 - control) had the highest iron content (3.92 mg/100g) while the 50:50 maize:pigeon pea flour (SMF6) had the lowest iron content (2.16 mg/100g) and there were significant differences amonth the six samples (Table 3). It was observed that the iron content significantly decreased as the percentage of maize flour decreased and the percentage of pigeon pea flour increased. The reduction in the iron values during the substitution of maize with pigeon pea indicates the unsuitability of pigeon pea flour to enhance iron content of the flour blend. Iron is necessary for the synthesis of hemoglobin and myoglobin, as well as preventing anemia in minors and teenagers [23].

The calcium content of the breakfast cereal ranged from 27.95 mg/100g to 44.55 mg/100g. Breakfast cereal sample with 50:50 % maize: pigeon pea flour (SMF6) recorded the highest calcium content of 44.55 mg/100g, while 100:0 % maize: pigeon pea flour (SMF1-control) recorded the least calcium content of 27.95 mg/100g and significant differences were observed among breakfast cereals. However, there was no significant difference between the breakfast cereal sample produced with 90:10 - maize: pigeon pea flour (SMF2) and 80:20 - maize: pigeon pea flour (SMF3). Calcium content increased as pigeon pea flour was increased. Bolarinwa et al*.* [25] reported the calcium content in complementary food produced from the mixture of malted millet, plantain, and soybean flour in the range of 15.01–25.10 mg/100g which is less than that reported in this study. Calcium is essential for musculoskeletal systems [24].

The magnesium content of the breakfast cereal varied from 63.16 to 75.07 mg/100g. The breakfast cereal produced with 100 % flour (SMF1 - control) had the highest magnesium content (75.07 mg/100g) while the breakfast cereal sample with 50:50 - maize: pigeon pea flour (SMF6) had the least magnesium content of (63.16 mg/100g) and significant differences were observed among all the six breakfast cereals. The magnesium concentration dropped as the percentage of maize flour reduced while pigeon pea flour increased. The decrease in the magnesium content is a direct consequence of the reduction in the quantity of maize flour which has more magnessium. The magnesium content of breakfast cereals developed in this research was higher than the magnesium content of 44.26–51.49 mg/100g for a maize-based breakfast cereal made from blend of pea and anchote reported by Gemede [26]. Magnesium aids in the control of glycemic processes, neuro-muscular processes, and myocardial contraction [24].

### 3.4 Sensory quality of breakfast cereals made from maize-pigeon pea flour blend

The sensory scores for breakfast cereals produced with maize and pigeon pea flour blends are presented in Table 4.

**Table 4:** Sensory scores for breakfast cereals made from flour blends of maize and pigeon pea.

Samples	Maize: Pigeon Pea	Colour	Aroma	Taste	Texture	Overall Acceptability
SMF1	100:0	5.53^c^±0.99	4.87^b^ ± 0.92	5.53^b^ ± 1.30	5.53^c^±1.19	5.40^c^±0.99
SMF2	90:10	4.27^ab^ ± 1.49	3.93^a^±1.49	4.60^ab^ ± 1.60	4.27^b^ ± 1.87	4.40^b^ ± 1.30
SMF3	80:20	5.27^bc^±1.16	5.00^c^±0.93	4.80^b^ ± 1.27	4.87^bc^±1.06	5.07^b^ ± 1.03
SMF4	70:30	4.73^abc^±1.28	5.27^c^±0.88	5.27^b^ ± 0.96	4.73^bc^±1.28	4.80^b^ ± 1.15
SMF5	60:40	4.73^abc^±1.28	4.80^b^ ± 1.32	4.60^ab^ ± 1.81	4.87^bc^±0.99	4.53^b^ ± 1.55
SMF6	50:50	3.60^a^±2.29	4.33^b^ ± 1.95	3.60^a^±1.77	2.87^a^±1.46	3.40^a^±1.60

Sensory score values in the table are mean values ± standard deviation. Values in the same column bearing the different superscripts are statistically different at 5 % probability level.

#### 3.4.1 Colour

The panelists gave the colour of the breakfast cereal samples a sensory score ranging from 3.60 to 5.53 on 7-point Hedonic scale (Table 4). The breakfast cereal sample with 100:0 - maize: pigeon pea flour (SMF1 - control) had highest score of 5.53 (Fig. 1) while the 50:50 - maize: pigeon pea flour (SMF6) had the lowest score of 3.60 and significan differences were observed between brakfast cereals. (Fig. 6). The colour of the samples got darkened with an increasing inclusion of pigeon peas (Fig. 1, Fig. 2, Fig. 3, Fig. 4, Fig. 5, Fig. 6).

The mean score decreased significantly (p < 0.05) as the quantity of pigeon pea flour increased due to dark colour of pigeon pea flour. Pigeon pea contains polyphenolic compounds, which can undergo enzymatic oxidation when the cells are disrupted during processing resulting in the formation of dark pigments [27].

#### 3.4.2 Aroma

The mean scores ranged from 3.93 to 5.27 on a 7-point Hedonic scale. The score for the breakfast cereal with 70:30 % maize: pigeon pea flour (SMF4) was the highest (5.27), while the sample with 90:10 - maize: pigeon pea flour (SMF2) was the lowest (3.93). The aroma of the samples with 60:40 and 50:50 of Maize:pigeon pea were not significantly different while the rest differed significantly. As the amount of pigeon pea flour increased in the breakfast cereals, the aroma score significantly increased.

#### 3.4.3 Taste

The taste means scores ranged from 3.6 to 5.53 on a 7-point Hedonic scale. The breakfast cereal with 100:0 % maize: pigeon pea flour (SMF1 - control) had highest mean score (5.53) while the 50:50 % maize: pigeon pea flour (SMF6) had the lowest mean score (3.6) and were significantly different (p < 0.05). Generally, increasing pigeon pea flour resulted in a decrease in taste mean score due to increased intensity of beany flavor.

#### 3.4.4 Texture

The breakfast cereal with 100 % maize flour (SMF1-control) had the highest mean score for texture (5.53) while the breakfast cereal with 50 maize:50 pigeon pea flour (SMF6) had the lowest mean score for texture (2.87). While most of the breakfast cereals did not differ significantly, the texture of the breakfast cereal with 100 % maize flour (SMF1-control) differed significantly (p < 0.05) with the rest.

#### 3.4.5 Overall acceptability

The overall mean acceptability score of the breakfast cereals were in the range of 3.40–5.40 on 7-point Hedonic scale. The breakfast cereal sample with 100 % maize flour had the highest score of 5.4 while the breakfast cereal with 50 maize:50 pigeon pea flour (SMF6) had the lowest score of 3.4. The overall acceptability of the breakfast cereals were similar except for samples with 100 % and 50 % maize, respectively. The overall acceptability range in this study were lower than 4.91–7.45 range as previously reported by Edima-nyah et al. [4].

## 4 Conclusion

Maize and pigeon pea flour blend can be used to produce nutritious and acceptable breakfast cereals especially when maize substitution does not go beyond 30 %. Further incorporation of pigeon peas results into darker products that has low acceptability. Such product has increased crude fiber, protein, and ash content, but lower fat and carbohydrate content. Therefore, pigeon pea flour could be used as an important ingredient in improving the nutritional quality of breakfast cereals for consumption by both children and elderly population. This could therefore increase dietary intake of protein and minerals and contribute to eradication protein energy malnutrition and micronutrient difieciencies commom in developing counries. Efforts should therefore be made to diversify utilization of pigeon peas by incorporating in breakfast cereal formulations.

## CRediT authorship contribution statement

**Clement Chinedum Ezegbe:** Writing – original draft, Resources, Project administration, Methodology, Investigation, Formal analysis, Data curation, Conceptualization. **Smith G. Nkhata:** Writing – review & editing, Visualization, Validation, Supervision, Data curation. **Ekpeno Sunday Ukpong:** Conceptualization, Data curation, Validation, Writing – review & editing. **Mary Chikodili Ezeh:** Writing – review & editing, Supervision, Methodology, Investigation, Formal analysis. **Kalu Sunday Okocha:** Conceptualization, Data curation, Investigation, Supervision, Validation, Writing – review & editing. **Bernard Femi Pedro:** Writing – review & editing, Resources, Methodology, Investigation, Conceptualization.

## Ethics and consent

This study was reviewed and approved by the Department of Food Science and Technology dated 25th January 2024.

All participants provided written informed consent to participate in the study and for their data to be published.

## Ethics statement

This research was approved by the Department of Food Science and Technology, Faculty of Agriculture, Nnamdi Azikiwe University, and informed consent was obtained from the panelists who did the sensory evaluation.

## Data availability statement

Data included in the article/supplementary material is referenced in the article.

## Declaration of competing interest

The authors declare that they have no known competing financial interests or personal relationships that could have appeared to influence the work reported in this paper.
